# Supplementary material for: “I Did Not Believe You Could Get Better”—Reversal of Diabetes Risk Through Dietary Changes in Older Persons with Prediabetes in Region Stockholm
Source: Nutrients. 2019 Nov 4;11(11):2658. doi: 10.3390/nu11112658 (PMC6893725; doi:10.3390/nu11112658)
Supplement: Supplementary file 1 [file nutrients-11-02658-s001.pdf]

Supplementary material 1
A Social vignette

Imagine a woman who lives with a partner and teenage children. Over the years the family's diet has become more simplified consisting mainly of high calorie fast foods served with sweetened beverages. This person has recently gained a lot of weight and because she has a parent who died of complications from diabetes, she is now at risk for diabetes onset.

The woman decides to change the family's eating habits. She finds new recipes

for healthy food and buys home more vegetables than before. She surprises the family with food that has never been cooked in their kitchen, and she is very happy with the results. But the surprise was not positive for the family, who were very dissatisfied with the meals. They wanted food that they were familiar with and complained loudly. The atmosphere was unpleasant and there was a lot of uneaten food after the meals.

Later the same week, the woman prepares a new, healthy meal with a different recipe, but the meal is similar to one from the week before. No one likes the food and even the woman thinks that it was not as tasty as last time. In the evening, the person's partner brings chips and soda and everyone sits in front of the TV and they have quite a good time together. Even our imagined person eats snacks and drinks soda even if it doesn't feel right. Afterwards, she feels completely failed.

Questions:

- Do you have any thoughts about how you think it will be for this family in the future?
- Could the person have done something in a different way to change the eating habits?
- Can you think of any similar situations in your life or that of those around you?
- Do you recognize yourself in this situation somehow?
- How can you relate to this situation?
- Are you in a position in your family to control the purchase and planning of meals?
- How do you think it would have been for you if you were in the position of the imagined person?
